# The Design and Development of a Food Composition Database for an Electronic Tool to Assess Food Intake in New Caledonian Families

**DOI:** 10.3390/nu13051668

**Published:** 2021-05-14

**Authors:** Juliana Chen, Solène Bertrand, Olivier Galy, David Raubenheimer, Margaret Allman-Farinelli, Corinne Caillaud

**Affiliations:** 1 Discipline of Nutrition and Dietetics, Charles Perkins Centre, School of Life and Environmental Sciences, The University of Sydney, Camperdown, NSW 2006, Australia; margaret.allman-farinelli@sydney.edu.au; 2 Interdisciplinary Laboratory of Research in Education, University of New Caledonia, 98851 Noumea, New Caledonia; soleneb@spc.int (S.B.); olivier.galy@unc.nc (O.G.); 3 Pacific Community, 98800 Noumea, New Caledonia; 4 Charles Perkins Centre, School of Life and Environmental Sciences, The University of Sydney, Camperdown, NSW 2006, Australia; david.raubenheimer@sydney.edu.au; 5 Discipline of Biomedical Informatics and Digital Health, Charles Perkins Centre, School of Medical Sciences, Faculty of Medicine and Health, The University of Sydney, Camperdown, NSW 2006, Australia; corinne.caillaud@sydney.edu.au

**Keywords:** food composition database, dietary assessment, children, adults, Pacific Islands, Melanesia, Polynesia, mHealth, mobile applications, nutrition transition

## Abstract

The food environment in New Caledonia is undergoing a transition, with movement away from traditional diets towards processed and discretionary foods and beverages. This study aimed to develop an up-to-date food composition database that could be used to analyze food and nutritional intake data of New Caledonian children and adults. Development of this database occurred in three phases: Phase 1, updating and expanding the number of food items to represent current food supply; Phase 2, refining the database items and naming and assigning portion size images for food items; Phase 3, ensuring comprehensive nutrient values for all foods, including saturated fat and total sugar. The final New Caledonian database comprised a total of 972 food items, with 40 associated food categories and 25 nutrient values and 615 items with portion size images. To improve the searchability of the database, the names of 593 food items were shortened and synonyms or alternate spelling were included for 462 foods. Once integrated into a mobile app-based multiple-pass 24-h recall tool, named iRecall.24, this country-specific food composition database would support the assessment of food and nutritional intakes of families in New Caledonia, in a cross-sectional and longitudinal manner, and with translational opportunities for use across the wider Pacific region.

## 1. Introduction

Countries and territories across the Pacific Islands face the triple burden of malnutrition from undernutrition, micronutrient deficiencies, as well as overweight and obesity, associated with non-communicable diseases (NCDs). From the Global Burden of Disease study, the prevalence of overweight and obesity across the Oceanic Pacific Island countries and territories are among the highest globally, for adults (as high as 88.3% for women), and for children (as high as 66.1% for girls) [1].

New Caledonia is a French overseas territory in the South Pacific with a population size of 271,407 [2]. The multiethnic society of New Caledonia is representative of the Pacific population, and consists primarily of Melanesians (indigenous Kanak people, 39.1%), Europeans (27.1%), Polynesians (8.2%), and Asians (2.7%) [3]. Studies in New Caledonia have found the BMI to be significantly higher among Melanesian and Polynesian adolescents as compared with those of European descent [4]. In particular, adolescent overweight was associated with Melanesian ethnicity, living in rural areas, and being of lower socioeconomic status [5]. Similar trends between ethnicity and weight have also been found to be present in adults [6].

NCDs associated with unhealthy diets, such as cardiovascular disease, diabetes, and cancer are the leading causes of mortality, contributing to over 80% of all deaths in the Pacific [7,8] and more than 50% of mortality in New Caledonia [9]. In Pacific communities, overweight and NCD progression have been accelerated by transitioning food environments facilitated by globalization and trade [10,11,12,13,14] combined with a decline in local agricultural and fishery practices [15]. Consequently, traditional nutrient-dense plant-based diets, particularly starchy tuber staples [16], have been replaced with imported energy-dense nutrient-poor processed foods and convenience fast foods high in saturated fat, added sugar, and/or added salt [8,14].

Nevertheless, nutrition transitions are poorly captured [17], and there is little understanding about specific foods and nutrients consumed by the New Caledonian population in general, let alone by ethnicity and geographic location. Our recent studies showed that both in rural and urban areas of New Caledonia, processed food is omnipresent in the diets of Melanesian adolescents, and that high proportions of children are drinking sugar sweetened beverages (SSBs) and energy drinks [16,18,19]. Trade data have revealed that SSBs [20] and processed foods make up the majority of imports to Pacific Island countries and territories [13] and New Caledonia [21]. The New Caledonian 2008 Household Expenditure and Income Survey [22] provides data on the inventory of foods purchased, produced, consumed away from home, and traded on a household level [17,23] but detailed information on the nutritional intake of individuals is required.

With the exception of Fiji [24], for the majority of low to middle-income countries in the Pacific Island countries and territories, including New Caledonia, no national nutrition survey has ever been conducted. With advancements in technology, smartphone nutrition applications (apps) for mobile devices are a convenient and valid way to survey diets at the population level [25,26,27,28,29]. Furthermore, electronic self-administered 24-h recall tools exist and have been validated for use in both children and adult populations (e.g., ASA-24 [30,31], Intake24 [32,33], and myfood24 [34,35]). However, none have been designed for the Pacific Island countries and territories, including New Caledonia. The backend to such dietary assessment tools for measuring and assessing diets is having a food composition database that reflects the food supply of the country [36,37]. The Food and Agricultural Organization (FAO) *Pacific Islands Food Composition Tables*, Second Edition was developed in 2004 [38], providing a nutrient composition data for close to 900 foods of the Pacific region. A feasibility study for updating to a third edition of the Pacific database was undertaken in 2019 with six countries, but no publicly available updates have been released and New Caledonia was not an included country in the scoping study.

New Caledonia was selected for this study, with its advantage of a representative Pacific population, and sectors of the population who may still follow a largely traditional Pacific lifestyle. The French and European influence of the New Caledonian food supply also provides a backdrop for assessing the impact of “Western” foods on the traditional diet. Therefore, this study aimed to develop a fit-for-purpose food composition database to analyze the food and nutritional intake of New Caledonian children and adults.

## 2. Materials and Methods

### 2.1. Context of the Electronic Tool

**Figure 1:**
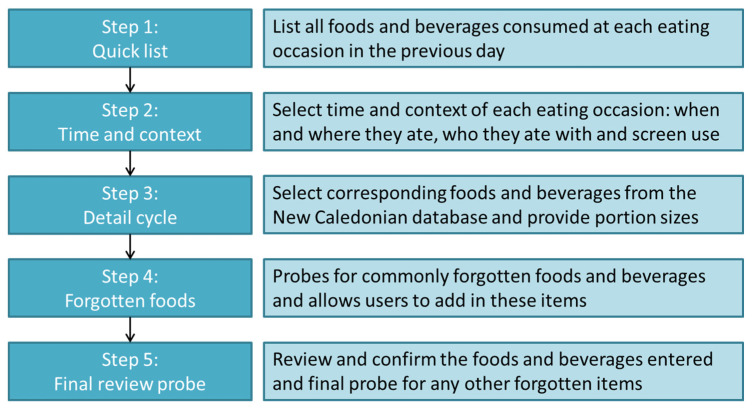
The multiple-pass 24-h recall process for the iRecall.24 app.

The New Caledonian food composition database described in the remainder of this paper was developed for integration into the backend of a mobile app-based 24-h recall tool, i.e., iRecall.24, that could be used to collect dietary information of children and adults. The iRecall.24 app functions in a similar way as other electronic self-administered 24-h recall tools (e.g., ASA-24 [39], Intake24 [40], and myfood24 [41]), whereby users undertake the multiple-pass 24-h recall process. The multiple-pass method involves users recalling their food intake over the previous 24 h according to five steps, as depicted in Figure 1. In the iRecall.24 app, firstly, the user provides a quick list of foods and beverages consumed at each eating occasion during the previous day. Details are collected about the time and context of each eating occasion including when and where they ate, who they ate with, and the presence of screen use (e.g., from television, smartphone, etc.). Then, users provide details about the foods and beverages consumed, by selecting corresponding foods from the New Caledonian database, estimating portion sizes of foods consumed, and reviewing items entered. Multiple probes are implemented in the app at each step to ensure that commonly forgotten foods and beverages (e.g., condiments, sweet and savory discretionary foods, soft drinks, and alcohol) have been captured and that users are able to add any additional items. Users are provided with a final opportunity to review and confirm the foods and beverages entered and a final probe for any other forgotten items is conducted. For the purposes of collecting dietary data in New Caledonia, the iRecall.24 app version 1.0 was developed in French, with corresponding French language version of the New Caledonian food composition database used. The development of the iRecall.24 app is described elsewhere.

### 2.2. Database Development

The FAO *Pacific Islands Food Composition Tables*, Second Edition, 2004 (Pacific database) [38] formed the foundation for the development of this food composition database for New Caledonia. A collaboration between accredited New Caledonian and Australian dietitians and other researchers modified the original Pacific database to ensure it captured the contemporary food environment in New Caledonia, thereby enabling measurement of nutrients from food and beverage (hereafter referred to as food) intake and for future longitudinal assessment of the nutrition transitions among the New Caledonian population. This database was compiled originally in English, but food names and modifications were completed in French for uploading into the iRecall.24 app. The *Tables de composition des aliments du Pacifique*, developed by The Pacific Community in 2003 [42], was used for corresponding French food name translations to those in the Pacific database.

**Figure 2:**
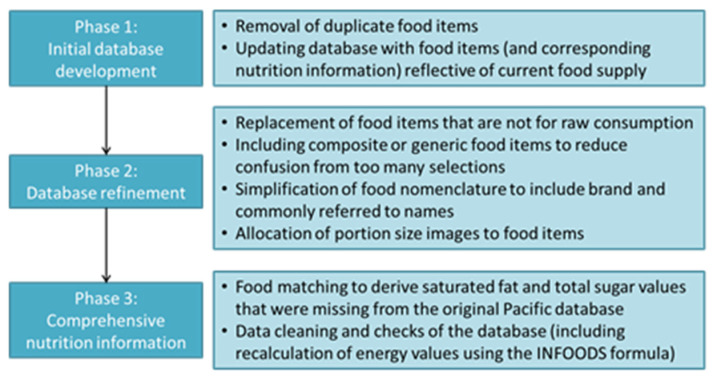
Phases in the development of the New Caledonian food composition database to support the iRecall.24 app.

This New Caledonia food composition database was developed in three phases (as summarized in Figure 2 below): Phase 1, initial database development to expand the range of foods; Phase 2, refinement of database items and naming and assignment of portion size images for food items; Phase 3, imputation of missing nutrient values for food items, with a focus on saturated fat and total sugar.

#### 2.2.1. Phase 1: Initial Database Development

Initial cleaning of the Pacific database was carried out to remove any duplicate food items. The original Pacific database was expanded to better reflect the current food supply in New Caledonia. The original Pacific database had not been updated in more than 15 years, and therefore it was necessary to update food items to reflect the contemporary food environment associated with the nutrition transition.

##### Sourcing of Food Items

With no previous national nutrition survey to provide guidance on foods consumed by the New Caledonian population, additional food items for inclusion were gathered from supermarket inventories, visits to local food outlets, and local practicing dietitians with knowledge of traditional recipes and specialty items commonly consumed.

A list of branded items for sale in New Caledonia was obtained from the websites of major supermarkets. From this, 6163 were identified as food items. Nutritional supplements and weight loss products were excluded. The food items were coded into 66 categories. Food item categories determined from the supermarket data were compared to those of the individual food items and categories in the Pacific database. Where it was identified that there was inadequate representation of the food category or specific items, these were added into the database.

Fieldwork in New Caledonia by the Australian and local French-speaking dietitians (J.C. and S.B.), included visits to two weekend markets, six food stalls/trucks, two popular takeaway food stores, a local school cafeteria, and three local supermarkets. According to the food categories and food items from the original Pacific database, the dietitians checked and confirmed the availability of these foods during the field visits, or collected additional details on the variety, supply, and range of other local food options if they were identified as absent in the original Pacific database. Another local dietitian (E.S.) working in New Caledonia as part of the research team contributed to a list of commonly consumed foods based on their experience with local clients and it was ensured that these food items were reflected in the database. Traditional dishes and local specialty foods that were not present in the original Pacific database were also reviewed by J.C. and S.B. and included from the *Food Portions book for French Polynesia* developed by the Ministry of Health, Department of Health; the House for Diabetes, Centre for Therapeutic Education; and the French Polynesian Dietitian’s Association in Papeete [43].

##### Sourcing Food Composition Data for Additional Food Items Identified

Nutrition information for these additional new food items were gathered from the English edition of the ANSES-CIQUAL French Food Composition Tables, version 2017 (CIQUAL) [44]; the Australian Food, Supplement, and Nutrient Database (AUSNUT) 2011–2013 [45]; the 2018 New Zealand FOODFiles database [46]; and the U.S. Department of Agriculture (USDA) FoodData Central 2019 [47]. The country of origin of the food item and matching based on food descriptors were considered to determine which food composition data source was selected. Food labelling information was also used to derive nutrition information for popular items in New Caledonia where the food was not available in the databases (e.g., Maggi seasoning sauce). For mixed dishes in the French Polynesia portions book, nutrition information were calculated based on ingredient lists from recipe formulations provided with this resource [43]. Nutrient information for individual ingredients were derived primarily from the original Pacific database, as well as the CIQUAL [44] or AUSNUT [45] databases.

#### 2.2.2. Phase 2: Refinement Based on Feedback

##### Usability Testing

The French version of the database from Phase 1 was imported into the backend of the purpose-designed iRecall.24 app, for a self-administered 24-h recall. Trialing the app allowed users to experience the range of foods and names of foods and portion sizes in the food composition database. Adopting a ”think aloud” approach for usability testing [48], users from our research team, including three dietitians local to New Caledonia or from the French Island territories, Wallis, and Futuna; two researchers; and two developers from the app company tested the app in French and provided feedback on the database-related functionality of the app.

On the basis of the feedback, food items that were raw or would not be consumed in their uncooked state were removed and replaced by cooked varieties. It was also evident from usability testing that users did not necessarily know about specific varieties of foods (especially for fruits, vegetables, and seafood). To minimize confusion about which food item to select when accessing the database within the iRecall.24 app, different varieties or cultivars of the same food (e.g., for jackfruit, *A. heterophyllus*, and *A. integer*) were combined into a single item, either by replacement with an existing composite or generic item from the original Pacific database or by averaging nutrient compositions. The FAO/INFOODS Guidelines for Food Matching Version 1.2 [49] was used to guide this process. Food items that were unavailable or unlikely to be consumed in New Caledonia were removed. This method has been used in the development of other nutrition databases [36] and is supported by qualitative findings that support the usability of food databases within nutrition apps [36,50,51].

##### Modifying Food Nomenclature

The naming of food items within the Pacific database, and other database sources (e.g., CIQUAL) were quite detailed to capture the taxonomic or scientific naming of certain species of foods (e.g., green leaves and fish and seafood) as well as cooking methods. Consequently, the French names of foods were shortened and simplified from the long string generic names to improve searchability within the iRecall.24 app. This involved, simplifying specific descriptors of the content of the food items, such as the specific ingredients within the food, particularly if there was only one common option available in New Caledonia. Where one food item description included multiple cooking methods, such as “boiled, microwaved, steamed, or poached”, the most common method used in New Caledonia was chosen. The modification of the food nomenclature was guided by the food and meal preparation knowledge the French-speaking dietitian local to New Caledonia (S.B.).

Where food items had been derived from English databases (e.g., AUSNUT, FOODFiles, and USDA FoodData Central), Google translate was used for translations into French, and then simplified. Synonyms and alternate spellings were added to food items where appropriate to improve the ability to locate food items consumed.

All modifications to the database and French naming of foods were reviewed and checked by two independent French speaking members of our research team (S.B. and C.C.), one being a French dietitian with public health and community experience in improving nutrition with Pacific Island communities.

##### Portion Size Images

**Figure 3:**
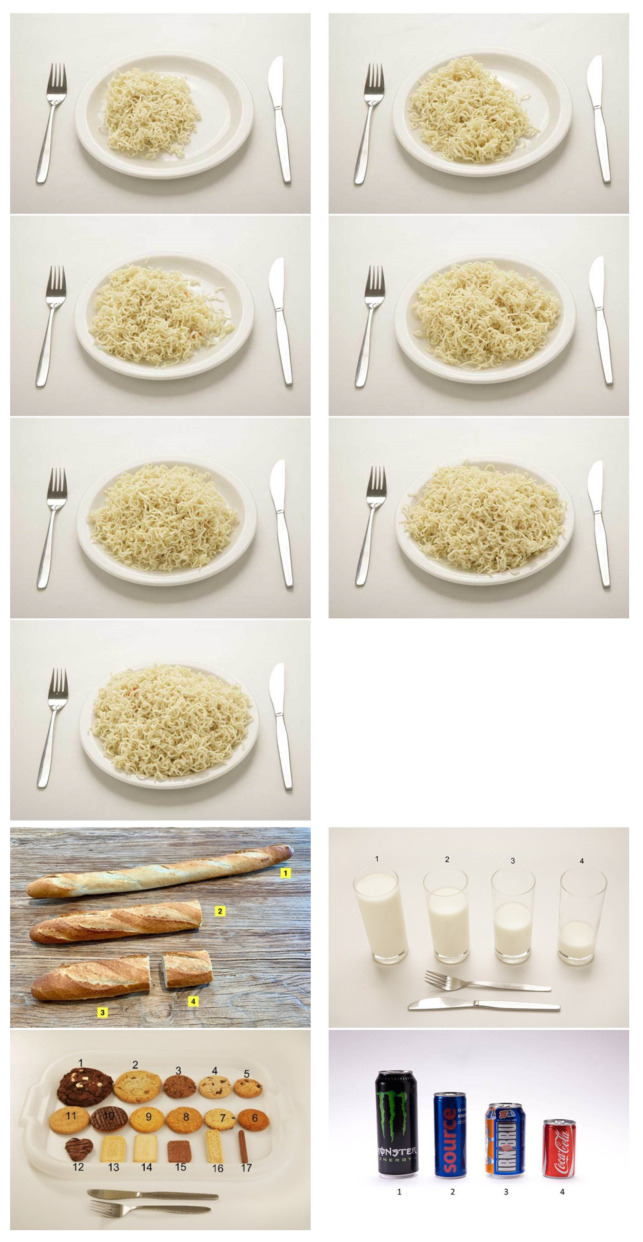
Formats of portion size images for foods and beverages, as served or in a group.

All food items (food and beverages) in the New Caledonian database were assigned portion size images and/or household or gram measures. Portion size images were available in two formats for users to select from (Figure 3): (1) as served on a plate with three to seven images options corresponding to different amounts of food (e.g., noodles) or (2) in a range of sizes (e.g., different lengths of baguette or different volumes of a beverage in a glass) or in a group with different varieties (e.g., assorted biscuits or soft drink/energy drink cans).

#### 2.2.3. Phase 3: Ensuring Comprehensive Nutrition Information

##### Saturated Fat and Total Sugar Nutrient Information

The original Pacific database consisted of food composition values for energy including fiber (in kilojoules (kJ) and kilocalories (kCal)), water (g), protein (g), total fat (g), carbohydrate (g), total dietary fiber (g), cholesterol (mg), as well as 15 vitamins and minerals (sodium (mg), magnesium (mg), potassium (mg), calcium (mg), iron (mg), zinc (mg), retinol (µg), beta-carotene equivalents (µg), total vitamin A equivalents (µg), thiamin (mg), riboflavin (mg), niacin (mg), vitamin B12 (µg), vitamin C (mg), and Vitamin E (mg)). However, saturated fat and total sugar were not included. Given the nutrition transition occurring in the Pacific Island region, it was deemed important to include these nutrients in this New Caledonian database. The FAO/INFOODS Guidelines for Food Matching [49] was again used to determine appropriate food matches from which saturated fat and total sugar values could be derived. This involved food identification based on food name and descriptor and assessing water and total fat and carbohydrate content.

Where available, the nutrient data for the source codes in Appendix VI, food index of the Pacific Islands food composition tables [38] were used to derive saturated fat and total sugar values of foods from the Pacific database. Saturated fat (g) and total sugar values (g) were calculated proportionally to the total fat and carbohydrate values of the matched food item, unless there was an exact match between food items (i.e., the food and all its descriptors in the source codes match exactly with the food and all its descriptors from the Pacific database) and the total fat and carbohydrate values were identical. This method was chosen over replacing all nutrient values to retain as much of the original dataset which included Pacific Islands-generated analytical data.

If the original publication of the source codes could not be located or there was no inclusion of saturated fat and total sugar values, data were borrowed and imputed in a proportional manner based on the total fat and carbohydrates from matching or similar foods or from data in scientific articles. Values from food packaging labels or calculations from ingredients in recipes were also used. Assumptions were also made, for example, where total fat or carbohydrate values were zero or where they would be not naturally occurring in a food, saturated fat and total sugar were assumed to be zero (e.g., no sugar in fresh meat, or no saturated fat in green leafy vegetables). The selection of the database for food matching was based on assessment of the quality of the food matching, the comprehensiveness of the nutrient data available, and the country most likely to supply that food to New Caledonia. Borrowed or imputed values came from the CIQUAL, AUSNUT, FOODFiles, USDA, and the Japanese food composition tables [52].

##### Data Cleaning and Checks

All energy values were recalculated based on the INFOODS formulas (which included energy from fiber) for kJ and kcal. Total vitamin A equivalents (retinol equivalents) were re-calculated using the INFOODS formula for all food items, as AUSNUT [53] uses a different formula to calculate provitamin A activity. For fruit and vegetable food items derived from the CIQUAL database, beta-carotene values were checked against a similar matched food from the original Pacific database or AUSNUT to confirm that values were not underestimating other beta-carotene equivalents. This was because the CIQUAL database only reported retinol and beta-carotene values, but not beta-carotene equivalents.

To improve the usability and ease of aggregated nutrient calculations for researchers or dietitians, where the Pacific or CIQUAL database had trace values (T, i.e., less than the limit of detection), these were replaced with zero values or nutrient values from matched food items from the AUSNUT or FOODFiles databases. Further data cleaning was conducted to remove the “<” sign in the CIQUAL database, and values were rounded down by 0.001, 0.01, or 0.1 decimal places, depending on the number of significant figures for that nutrient.

Data quality checks were carried out by two independent researchers, with any suspected errors reviewed against the original source food composition database.

## 3. Results

### 3.1. Phase 1

Table 1 presents the number of food items derived from different sources and their classification into the food categories across iterations during the development phases of the New Caledonian food composition database. The original Pacific database consisted of nutrition composition data for 889 foods in 20 food categories. Nine items identified as duplicates were removed. Then, 644 items were added, of which 74% (*n* = 476) were from the CIQUAL database; 14% (*n* = 93) from AUSNUT; 9% (*n* = 58) from the French Polynesia portions book contributing to traditional dishes and local food specialties; 1.2% (*n* = 8) from food packages/labels; and the remaining 1.4% (*n* = 9) from FOODFiles, USDA, and new generic foods or recipes plus a field for water (Table 1). Subsequently, at the end of Phase 1, the New Caledonian food composition database was expanded to a total of 1524 food items.

The original 20 food categories were expanded into 41 categories (Table 1), particularly with cereal and cereal products expanded into four categories, meat and poultry into two categories, milk and milk products into four categories, confectionery into four categories, herbs and spices into two categories, and beverages into six categories. The original processed food category was replaced with four new categories “chips and savory snacks”, “pizzas, pies and burgers”, “mixed canned foods”, and “soup”. New categories of “sandwich” and “savory canapes” were added. These changes were made to improve distinction of processed and discretionary foods from those of the core food groups.

### 3.2. Phase 2

In Phase 2, the main problems encountered during usability testing were around foods not found by users as they were missing from the database, or appeared in the database as a different name to what the users referred to them as (e.g., “pain au chocolat” instead of “chocolatine”), or users had entered the name of the food category instead of specific type of food (e.g., “viande” (meat) instead of beef, pork, chicken). Table 2 presents the other issues relating to database searching, including too many search returns or confusion over different varieties of foods. Spelling issues also affected the success of searches including, misspelling or variations in spelling, the presence or absence of French accents and special characters (e.g., “oeuf” instead of “œuf”), or use of plurals or singular spelling (e.g., “chou-fleur” vs. “choux-fleur”).

On the basis of the feedback, three main areas were identified for improvements. The database was expanded by an additional 185 foods to include the missing food items and additional common local foods or traditional dishes. Secondly, 737 food items and one food category (infant food and formula) were removed. This consisted of removal of the following: 422 items (57.3%) to reduce the number of food options; 80 items (10.9%) that were averaged or replaced with a composite or generic food item; 76 items (10.3%) that could not be eaten raw (e.g., starchy vegetables, some green leaves, dried legumes and lentils, raw meat and wild animal/game) or where the made up beverage option was already included (e.g., coffee or cocoa powder); 69 items (9.4%) that were not commonly available in New Caledonia; 38 items (5.1%) were baby foods (e.g., breastmilk, formula, and baby foods); 37 items (5%) were replaced with another matching food item where nutrient information was comprehensive; and 15 items (2%) which were already represented or a duplicate of another food item. The final version 1.0 of the New Caledonian food composition database contained a total of 972 food items, with 40 food categories (see Table 1, phase 2).

Finally, the names of 593 food items (61%) were simplified and shortened or edited (e.g., using brand names rather than long descriptors, for instance,“biscuit au chocolat, fourré à la crème (Oreo)” was changed to just the brand name “Oreo”), and synonyms or alternate spelling were included for 462 items (47.5%). Fuzzy string matches were employed within the revisions of the iRecall.24 app to enhance searchability by allowing for minor variations in spelling to account for misspelling, singular and plurals, accents, and spaces.

A total of 353 unique food or assorted food group portion size images were included in this New Caledonian food composition database. The same portion size images could be used for similar foods (e.g., images of brie cheese used for other soft cheeses) and ”group” layout pictures could contain between two and 23 assorted food options (e.g., different types or brands of confectionery). Therefore, the portion size images were coded to 615 (63.3%) of the 972 food items. Portion size images were sourced via open source from the online 24-h recall tool, Intake24 [54] (48.7%, *n* = 172); the French Polynesia portions book (45%, *n* = 159); and additional images for 22 unique foods were taken, particularly for green leafy vegetables, cheeses, processed meats, and baguette.

### 3.3. Phase 3

In phase 3, complete saturated fat and total sugar values were included for all 972 food items in version 1.0 of this New Caledonian database. For 456 (47%) food items, the saturated fat and/or total sugar values were derived from the original source of data for those items; 56 (5.7%) food items were calculated from recipes or scientific articles for saturated fat and 52 for total sugar; two food items were derived from food labels (0.2%); and 31 food items with assumed saturated fat values and 42 for total sugar (Table 3). The remaining 516 foods required matching with food items from other food composition database sources to determine the most appropriate saturated fat and total sugar values to use. Exact matches for saturated fat were present in 159 food items, while 122 foods had exact matches for total sugar, with the majority of matches coming from AUSNUT.

## 4. Discussion

We developed a comprehensive country-specific food composition database for New Caledonia, to be integrated into a mobile app-based multiple-pass 24-h recall tool iRecall.24. Version 1.0 of this New Caledonian database comprises a total of 972 food items covering 40 food categories and 25 nutrient values including saturated fat and total sugar. To assist with estimation of portion sizes within the iRecall.24 app, 615 food items have associated portion size images. Usability testing allowed for improvements in the searchability of the database, through refinement of the food items included, but also of food naming and spelling.

Country-specific databases have been emphasized as an important feature of the usability and accuracy of app-based dietary assessment tools, for researchers and their study population, as well as health care professionals, such as dietitians and their clients in the general public [36,37,50,55]. To reflect the changing food supply in New Caledonia, food composition data were drawn from the databases of multiple countries based on the country of origin of the imported food item. Branded and generic food items, as well as local or traditional recipes and food items, were also included within the database, with the aim of increasing the relevance of search results in the iRecall.24 app to the New Caledonian population and improve accuracy in measurement of food and nutritional intake. These methods are in alignment with the development of other databases for electronic dietary assessment tools, such as the EaT app to assess dietary intake in Australian young adults [36], as well as other online self-administered 24-h recall tools, myfoods24 [37] and Intake24 [40].

Another key focus in developing this database was to increase representation of imported processed foods. This new food composition database for New Caledonian provides access to an expanded number of processed and discretionary food items and food categories, and importantly saturated fat and total sugar values have been included as part of the nutrient data. These database considerations are particularly relevant given the prevalence of processed foods across Pacific Island countries and territories and in New Caledonia [13,14] and the diet-related NCDs associated with nutrition transitions [11]. While a previous systematic review found a high contribution of fat (particularly saturated fat), sugar, and salt in the diets of many Pacific Island countries and territories [56], the study also noted the general lack of dietary data across the Pacific and New Caledonia, and concluded that higher quality research using reliable and valid dietary assessment tools was required. High unhealthy food consumption with 27% of daily food intake was recently observed in Melanesian adolescents from New Caledonia. These dietary changes might explain the high percentage of overweight and obesity (38.1% for rural and 31.7% for urban adolescents) observed in this study, therefore, further comprehensive investigation of dietary intakes is needed [16]. In the future, the food and nutrient data within this database will be validated, in the context of the iRecall.24 app against 24-h recalls in children and adults in New Caledonia.

As with other electronic self-administered 24-h recall tools [39,40,41,57], portion size images have been coded to food items in this database, for display in the iRecall.24 app. The benefit of portion size images is that they can assist users in estimating the amount of food consumed, and therefore increase the accuracy of self-reported dietary intake data [57]. Portion size images attached to this database were derived from Intake24 [40], which sourced its images from the *Young Person’s Food Atlas* that has been validated in children 4–16 years old [58,59]. The images from the French Polynesia portions book and the new images taken require validation in New Caledonian children and adults in future studies.

Further usability testing is underway for the database via integration with the iRecall.24 app. The app and database will be piloted with adults and children in the field in local New Caledonian communities using similar methods adopted in field testing of other digital 24-h recall tools [60]. Users will be asked to provide feedback on the usability of the system, including completion of the system usability scale [61]. Assessments of the functionality of the database and app will be compared between participants self-administering the app versus dietitians administering the app with participants.

Application of this food composition database in the iRecall.24 app would allow greater ease of conducting assessments of individuals’ usual intake as well as regional or national nutrition surveys in New Caledonia. The contribution of processed and discretionary foods to traditional diets and nutrient intake over time can be monitored in the nutrition transition. Furthermore, in the context of public health, governments can utilize this data to inform targeted health policies and nutrition interventions to meet World Health Organization global targets for the prevention and control of NCDs [8].

### Limitations and Strengths

The main limitation in the development of this New Caledonian database is that nutrient values for added items were derived from other food composition databases, imputed from other foods, or calculated based on recipes. While some of these data come from the original chemically analyzed source food composition database, direct laboratory analysis of the foods imported into New Caledonia and local or traditional foods have not been conducted. Direct chemical analysis of these food items, including saturated fat and other fatty acids, and total sugar and added versus free sugars, should be considered a priority in future updates to the database for a better understanding of micronutrient deficiencies and under- and over-nutrition and their impact on population health in New Caledonia. Drawing upon an Italian example [62], analyzing other bioactive components such as nutrient antioxidants in traditional foods and recipes may also be beneficial for greater advocacy of consuming traditional diets for NCD prevention and cultural preservation.

As no national nutrition survey has been conducted in New Caledonia and understanding about the range of foods consumed by the New Caledonian population is limited, it is possible that there are some common foods missing from this version of the database. With ever-changing product reformations and new food developments, keeping food composition databases up-to-date is a recognized challenge, even among countries, such as the UK, with well-established food composition databases [63]. Nevertheless, a strength of version 1.0 of this New Caledonian food composition database is that it has been structured in a dynamic manner that will allow for future updates and further refinements to the database once field testing has been conducted. As food consumption data are gathered, this will support additional modifications to ensure that popular foods and recipes are adequately represented in the database, and adjustments to the database can be made to include specific varieties or cultivars of food items based on popularity of intake in New Caledonia. While the food represented in this database is primarily adapted for use in New Caledonia and other French territories, there is flexibility within the database to also update the source and nutrient values based on changes in patterns of trade, product reformulations, or as updates to the original Pacific database occur. A similar process can be undertaken to personalize, adapt, and extend the database for use in other Pacific Island countries and territories, and therefore increase the translational potential of this database.

## 5. Conclusions

In this paper, we described the development of a food composition database that can be integrated into a mobile app-based 24-h recall tool iRecall.24. The database offers flexibility for future refinement of the food and nutrient data according to changes in the food supply. With its existing overlap with the broader *Pacific Islands Food Composition Tables*, this database has translational capacity for adaptation to capture dietary data across the wider Pacific region. Future research to validate the iRecall.24 app and associated food composition database and portion size images in the New Caledonian population will ensure that a robust dietary assessment tool is available for use by dietitians, researchers, and government agencies. The tool has potential to enable accurate assessment of individual and population intakes and with nutrition transitions underway, improve monitoring of changes in dietary patterns and food and macro- and micronutrient intake, including over- and under-consumption. Such data and evidence can provide the necessary evidence to develop targeted public health policies and individual and community-level nutrition intervention for children and adults in New Caledonia, to improve diets and reduce diet related health risks. 

## Figures and Tables

**Table 1:** Sources and categories of food items during the development phases of Version 1.0 of the New Caledonian food composition database.

	Source								
Food Category	PIFCT	CIQUAL	AUSNUT	FP Portions Book	New Zealand FOOD Files	USDA	Food Package Label	New Generic Food or Recipe	Total Items by Category
Starchy staples									
Original PIFCT	71								71
Phase 1	69	4	0	1	0	0	0	0	74
Phase 2	22	1	6	1	2	2	0	2	36
Cereal and bread									
Original PIFCT	81 ^1^								81
Phase 1	47	13	14	0	0	0	0	0	74
Phase 2	29	11	10	0	0	1	0	1	52
Crackers and crispbread									
Original PIFCT	N/A ^2^								N/A
Phase 1	2	14	2	0	0	0	0	0	18
Phase 2	0	5	7	0	0	0	0	0	12
Cookies and biscuits									
Original PIFCT	N/A ^2^								N/A
Phase 1	9	25	2	0	0	0	0	0	36
Phase 2	8	18	4	0	0	0	0	0	30
Pastries and cakes									
Original PIFCT	N/A ^2^								N/A
Phase 1	31	25	3	16	0	0	0	0	75
Phase 2	21	21	5	16	0	0	0	0	63
Green leaves									
Original PIFCT	66								66
Phase 1	66	4	4	1	0	0	0	0	75
Phase 2	42	1	8	0	0	0	0	1	52
Other vegetables									
Original PIFCT	73								73
Phase 1	70	16	2	0	0	0	0	0	88
Phase 2	42	7	21	0	0	0	0	0	70
Fruit									
Original PIFCT	87								87
Phase 1	87	7	4	0	0	1	0	0	99
Phase 2	49	4	3	0	0	1	0	0	57
Nuts and seeds									
Original PIFCT	34								34
Phase 1	34	0	5	0	0	0	0	0	39
Phase 2	14	0	7	0	0	0	0	0	21
Legume and legume products									
Original PIFCT	33								33
Phase 1	35	19	0	0	0	0	0	0	54
Phase 2	16	10	1	0	1	0	0	0	28
Fish									
Original PIFCT	44								44
Phase 1	44	18	3	5	4	0	0	0	74
Phase 2	30	13	12	3	5	1	0	0	64
Shellfish and seafood									
Original PIFCT	39								39
Phase 1	39	2	5	2	0	0	0	0	48
Phase 2	27	4	3	2	0	1	0	0	37
Meat and poultry									
Original PIFCT	84 ^1^								84
Phase 1	48	2	1	2	0	1	0	0	54
Phase 2	21	12	4	0	1	1	0	0	39
Processed and delicatessen meats & meat products									
Original PIFCT	N/A ^2^								N/A
Phase 1	29	31	0	2	1	0	0	0	63
Phase 2	12	17	0	1	1	0	0	0	31
Milk and milk products									
Original PIFCT	37 ^1^								37
Phase 1	12	3	6	0	0	0	0	0	21
Phase 2	4	3	6	0	0	0	0	0	13
Cheese									
Original PIFCT	N/A ^2^								N/A
Phase 1	14	49	3	0	0	0	0	0	66
Phase 2	7	31	2	0	0	0	0	0	40
Dairy desserts and cream									
Original PIFCT	N/A ^2^								N/A
Phase 1	10	2	2	0	0	0	0	0	14
Phase 2	4	4	3	0	1	0	0	0	12
Infant food and formula									
Original PIFCT	N/A ^2^								N/A
Phase 1	11	26	0	0	0	0	0	0	37
Phase 2	0	0	0	0	0	0	0	0	0
Eggs									
Original PIFCT	10								10
Phase 1	10	5	0	0	0	0	0	0	15
Phase 2	5	4	0	0	0	0	0	0	9
Fats and oils									
Original PIFCT	14								14
Phase 1	13	10	3	0	0	0	0	0	26
Phase 2	3	5	4	0	1	0	0	0	13
Processed foods ^3^									
Original PIFCT	54								54
Chips and savory snacks									
Original PIFCT	N/A ^2^								N/A
Phase 1	12	11	1	0	0	0	0	0	24
Phase 2	5	5	4	0	0	1	0	0	15
Pizzas, pies and burgers									
Original PIFCT	N/A ^2^								N/A
Phase 1	14	23	0	0	0	0	0	0	37
Phase 2	6	19	0	0	0	0	0	0	25
Mixed canned foods									
Original PIFCT	N/A ^2^								N/A
Phase 1	5	2	0	0	0	0	0	0	7
Phase 2	1	2	0	0	0	0	0	0	3
Soup									
Original PIFCT	N/A ^2^								N/A
Phase 1	4	29	1	0	0	0	0	0	34
Phase 2	1	8	6	0	0	0	0	0	15
Sandwich									
Original PIFCT	N/A ^2^								N/A
Phase 1	1	25	0	4	0	0	0	0	30
Phase 2	1	15	4	4	0	0	0	0	24
Mixed cooked dishes									
Original PIFCT	20								20
Phase 1	17	17	6	18	0	0	0	1	59
Phase 2	11	10	13	14	0	0	0	1	49
Savory canapes									
Original PIFCT	N/A ^2^								N/A
Phase 1	0	6	1	5	0	0	0	0	12
Phase 2	0	4	1	5	0	0	0	0	10
Confectionery									
Original PIFCT	26 ^1^								26
Phase 1	11	17	2	1	0	0	0	0	31
Phase 2	6	10	3	1	0	0	0	0	20
Chocolate									
Original PIFCT	N/A^2^								N/A
Phase 1	6	19	0	0	0	0	0	0	25
Phase 2	6	10	1	0	0	0	0	0	17
Spreads									
Original PIFCT	N/A ^2^								N/A
Phase 1	9	7	0	0	0	0	0	0	16
Phase 2	4	1	2	0	0	0	0	0	7
Nut and cereal bars									
Original PIFCT	N/A ^2^								N/A
Phase 1	2	6	0	0	0	0	0	0	8
Phase 2	0	3	0	0	0	0	0	0	3
Herbs and spices									
Original PIFCT	47 ^1^								47
Phase 1	28	0	0	0	0	0	0	0	28
Phase 2	20	0	4	0	0	0	0	0	24
Condiments, sauces and dressings									
Original PIFCT	N/A ^2^								N/A
Phase 1	19	4	2	0	0	0	2	0	27
Phase 2	9	3	5	0	0	0	2	0	19
Beverages ^3^									
Original PIFCT	37								37
Beverage—alcoholic									
Original PIFCT	N/A ^2^								N/A
Phase 1	15	2	0	0	0	0	0	0	17
Phase 2	8	1	2	0	0	0	0	0	11
Beverage—coffee, tea, cocoa									
Original PIFCT	N/A ^2^								N/A
Phase 1	11	21	1	0	0	0	0	0	33
Phase 2	2	6	3	0	0	0	0	0	11
Beverage—fruit concentrate, fruit drink, cordial									
Original PIFCT	N/A ^2^								N/A
Phase 1	3	4	7	0	0	0	6	0	20
Phase 2	0	5	0	0	0	0	0	0	5
Beverage—Juice									
Original PIFCT	N/A ^2^								N/A
Phase 1	4	0	0	0	0	0	0	0	4
Phase 2	3	0	3	0	0	0	0	0	6
Beverage—soft drink									
Original PIFCT	N/A ^2^								N/A
Phase 1	5	5	6	1	0	0	0	0	17
Phase 2	4	2	4	0	0	0	0	0	10
Beverage—water									
Original PIFCT	N/A ^2^								N/A
Phase 1	3	1	5	0	0	0	0	1	10
Phase 2	2	2	1	0	0	0	0	1	6
Coconut products									
Original PIFCT	14								14
Phase 1	10	0	2	0	0	0	0	0	12
Phase 2	4	0	2	0	0	0	0	0	6
Wild animals/game									
Original PIFCT	21								21
Phase 1	21	2	0	0	0	0	0	0	23
Phase 2	6	1	0	0	0	0	0	0	7
Phase 1 total items by source	880	476	93	58	5	2	8	2	1524
Phase 2 total items by source	455	278	164	47	12	8	2	6	972

Note: PIFCT, *Pacific Islands Food Composition Tables,* Second Edition [38]; CIQUAL, ANSES-CIQUAL 2017 database [44]; AUSNUT, Australian Food, Supplement and Nutrient Database (AUSNUT 2011–2013) [45]; FP portions book, food portions book for French Polynesia [43]; New Zealand FOODFiles, New Zealand FOODFiles 2018 database [46]; USDA, U.S. Department of Agriculture FoodData Central 2019 [47]; N/A, not applicable; ^1^ the total number of items present in the original category of the *Pacific Islands Food Composition Tables,* Second Edition; ^2^ new category created in this New Caledonia food composition database; ^3^ original category in the *Pacific Islands Food Composition Tables*, Second Edition, but replaced by new sub-categories in this New Caledonia food composition database.

**Table 2:** Feedback provided on the database during usability testing and the actions for improvement.

Issue	Food Items (Corresponding Food in the Database)	Improvement Actions
Not found—missing item	Salade verte Frites de pomme de terre, maison Cotelette de porc Jus d’orange pressé Chocolat au lait Vin rosé Quiche aux épinards	Food items added
Not found—different name or the name of a food category	Baguette blanche (Pain dit à la française/à l’italienne) Soupe chinoise (Soupe asiatique) Viande (Boeuf; porc; poulet; etc.) Chocolatine (Pain au chocolat)	Food name refined Synonyms added to the database
Not found—too many search results	Mozzarella (Fromage type mozzarella)	Food name refined Food category tags included as a synonym rather than in the main name
Not found—alternate or incorrect	Olives noire *(Olive)* Olives vertes *(Olive)* Yaourt *(Yogourt)* Brocolis *(Broccoli)* Choux-fleur *(Chou-fleur)* Oeuf *(Œuf)* Boeuf *(Bœuf)* Redbull *(Red bull)* Pome de terre *(Pomme de terre)* Iniame *(Igname)* Pin *(Pain)* Gato *(Gâteau)* Musli *(Muesli)* Ri, ris *(Riz)* Epinar *(Épinard)* Conconbre *(Concombre)* Carote *(Carotte)* Aricot *(Haricot)* Ton *(Thon)* Socisse *(Saucisse)* Conté *(Comté)* Piza *(Pizza)*	Alternate spelling (including variations in accents) added as synonyms Fuzzy logic applied for searches in app coding to allow for slight variations in spelling, accents, punctuation, spaces and plural and singular forms of words
Confusing items	Not knowing what “Lait condensé” was Querying relevancy of baby/infant food “Lait maternel, colostrum, 1er âge, 2eme âge” Querying relevancy of items that would not be consumed raw (e.g., saucisse, crue—sausages raw) Not knowing the difference between pineapples from different countries (e.g., Ananas (d’Australie) vs. Ananas (de Papouasie-Nouvelle-Guinée)) Not knowing how foods were cooked or what ingredient was used (e.g., Bœuf, viande hachée: ordinaire, mijotée, égouttée)	Irrelevant items removed Average and merge similar items together to reduce options Food name refined and descriptions simplified

**Table 3:** Data sources for saturated fat and total sugar values for food items (*n* = 972) in the New Caledonian food composition database.

Source	Saturated Fat	%	Total Sugar	%
CIQUAL				
Original source value	277	28.5	275	28.3
Exact food match value	4	0.4	5	0.5
Proportional value	52	5.3	47	4.8
Imputed proportional value	2	0.2	2	0.2
Averaged value	4	0.4	4	0.4
AUSNUT				
Original source value	161	16.6	161	16.6
Exact food match value	140	14.4	110	11.3
Proportional value	146	15.0	178	18.3
Imputed proportional value	19	2.0	20	2.1
Averaged value	6	0.6	6	0.6
New Zealand FOODFiles				
Original source value	12	1.2	12	1.2
Exact food match value	10	1.0	5	0.5
Proportional value	23	2.4	28	2.9
Imputed proportional value	2	0.2	2	0.2
USDA FoodData Central				
Original source value	6	0.6	8	0.8
Exact food match value	4	0.4	2	0.2
Proportional value	12	1.2	9	0.9
Japanese food composition tables				
Exact food match value	1	0.1	0	0.0
Proportional value	2	0.2	0	0.0
Calculated from FP portions book recipe	48	4.9	48	4.9
Calculated from other recipes	6	0.6	6	0.6
Calculated from scientific article	2	0.2	0	0.0
Food packaging label	2	0.2	2	0.2
Assumed value	31	3.2	42	4.3
Total food items	972	100	972	100

FCDB, food composition database; CIQUAL, ANSES-CIQUAL 2017 database [44]; AUSNUT, Australian Food, Supplement and Nutrient Database (AUSNUT 2011–2013) [45]; New Zealand FOODFiles, New Zealand FOODFiles 2018 database [46]; USDA, U.S. Department of Agriculture FoodData Central 2019 [47]; Japanese 2015 Food composition tables [52]; FP portions book, food portions book for French Polynesia [43].
